# Rediscovery of the critically endangered Hill's horseshoe bat (*Rhinolophushilli*) and other new records of bat species in Rwanda

**DOI:** 10.3897/BDJ.10.e83546

**Published:** 2022-05-19

**Authors:** Jon Flanders, Winifred F Frick, Julius Nziza, Olivier Nsengimana, Prince Kaleme, Marie Claire Dusabe, Innocent Ndikubwimana, Innocent Twizeyimana, Sospeter Kibiwot, Pierre Ntihemuka, Tina L Cheng, Richard Muvunyi, Paul Webala

**Affiliations:** 1 American Museum of Natural History, New York, United States of America American Museum of Natural History New York United States of America; 2 Tulane University, New Orleans, United States of America Tulane University New Orleans United States of America; 3 Bat Conservation International, Austin, United States of America Bat Conservation International Austin United States of America; 4 University of California Santa Cruz, Santa Cruz, United States of America University of California Santa Cruz Santa Cruz United States of America; 5 Gorilla Doctors, Kigali, Rwanda Gorilla Doctors Kigali Rwanda; 6 Rwanda Wildlife Conservation Association, Kigali, Rwanda Rwanda Wildlife Conservation Association Kigali Rwanda; 7 Centre de Recherches en Sciences Naturelles (CRSN), Lwiro, Democratic Republic of the Congo Centre de Recherches en Sciences Naturelles (CRSN) Lwiro Democratic Republic of the Congo; 8 Fauna and Flora International, Monrovia, Liberia Fauna and Flora International Monrovia Liberia; 9 University of Eldoret, Eldoret, Kenya University of Eldoret Eldoret Kenya; 10 Nyungwe National Park, Kitabi, Rwanda Nyungwe National Park Kitabi Rwanda; 11 Rwanda Development Board, Kigali, Rwanda Rwanda Development Board Kigali Rwanda; 12 Maasai Mara University, Narok, Kenya Maasai Mara University Narok Kenya

**Keywords:** Afromontane rainforest, Albertine Rift, Nyungwe National Park, Rwanda

## Abstract

**Background:**

For forty years, there has been growing uncertainty about whether Hill's horseshoe bat (*Rhinolophushilli*) still persists in Nyungwe National Park, Rwanda. Only known from one small area within the National Park, *R.hilli* is listed as Critically Endangered by the International Union for the Conservation of Nature (IUCN), based on its extremely small geographic range and presumed low number of mature individuals. Here, we present and describe bat species occurrence data contributed to the Global Biodiversity Information Facility (GBIF) that we collected as part of a long-term collaborative project to rediscover this lost species. This data paper describes the survey methods and findings resulting from cave roost surveys, capture surveys, and acoustic sampling of bat echolocation activity in Nyungwe National Park and surrounding areas in south-western Rwanda from 2013-2020 and their conservation relevance.

**New information:**

We report the discovery of an extant population of Hill's horseshoe bat (*Rhinolophushilli*) in Nyungwe National Park, Rwanda, 40 years since the last reported observation of the species in 1981. We also report the first record of Lander's horseshoe bat (*Rhinolophuslanderi*) in Nyungwe National Park and the first record of the Damara woolly bat (*Kerivoulaargentata*) in Rwanda. The dataset contributed to GBIF and described in this paper includes 278 occurrence records from 10 bat species of five families detected at 71 locations in or near Nyungwe National Park, Rwanda. We include a description of the morphological descriptions of *R.hilli* and present the first acoustic echolocation signatures and phylogenetic information for this species.

## Introduction

Nyungwe National Park in south-western Rwanda is one of the most biologically important montane rainforests in central Africa. The Park protects 1,019 km^2^ of montane rainforest, which is home to a diversity of wildlife species, including many species endemic to the Albertine Rift region of Africa (Rwanda Environment Management Authority 2015). The International Union for Conservation of Nature (IUCN) Red List of Threatened Species (Version 2021-3; www.iucnredlist.org) lists fifty-four species of bats as occurring in Rwanda, as well as additional, previously undocumented, species likely occur in Rwanda’s forest habitats. Habitat loss and fragmentation from activities, such as mining, logging, hunting, agriculture, and fires caused by wild honey collection, pose a significant threat to the region's biodiversity (Crawford 2012). The amount of intact forest in Nyungwe National Park has steadily reduced due to human encroachment and the forests surrounding the Park have disappeared almost entirely (Rwanda Environment Management Authority 2015). Due to the level of habitat change, the continued existence of rare and endangered species, such as the IUCN Critically Endangered Hill’s horseshoe bat (*Rhinolophushilli*), depends on accurate and up-to-date information being made available to resource managers who can utilise the data to implement effective conservation actions to protect species' at greatest risk of extinction.

### Hill's horseshoe bat (Rhinolophushilli)

First described by Aellen (1973), *Rhinolophushilli* was considered a synonym of *Rhinolophusruwenzorii* by Smith and Hood (1980), which they regarded as a subspecies of *R.maclaudi.* Csorba et al. (2003) considered *R.hilli* a subspecies of *R.ruwenzorii*, but Fahr et al. (2002) showed clear differentiation between the two species, re-instating *R.hilli* as a distinct species. Despite the activity determining its taxonomic status, *R.hilli* has only been observed on two occasions, once in 1964 and again in 1981, at two locations less than 8 km apart in the Uwinka region of Nyungwe National Park (Aellen 1973, Baeten et al. 1984). Basic knowledge about the species, such as where it roosts, population status, foraging habitat and behaviour and whether it can persist in degraded forests, is lacking. Based on what is known about closely-related species, *R.hilli* is presumed to be a cave-dwelling species that likely roosts in small colonies in forest caves (Fahr et al. 2002). *Rhinolophushilli* is believed to be severely threatened with extinction due to its limited geographic distribution, small population size, disturbance at caves and the degradation and loss of forest habitat in the Albertine Rift region (Webala et al. 2021). Caves within the boundaries of Nyungwe National Park were presumed to contain the last remaining populations of *R.hilli* (*Fahr et al. 2002*). Here, we present the results collected by our team as part of a multi-phased effort to conduct targeted, cost-effective surveys to prioritise species detection.

### Conservation relevance

Results of this work contribute to the overall knowledge of the biodiversity of the region and provide specific information to guide protective measures to save a critically-endangered bat from global extinction. Nyungwe National Park is one of the most biologically important montane rainforests in Central Africa. Documentation of the diversity of bat species occurring in the Park aids national Rwandan conservation efforts, which must balance competing interests. Since decades passed without any reported observation of *R.hilli*, uncertainty over its continued persistence impeded implementation of species-specific management actions, including safeguarding critical habitat for the species against encroachment or disturbance. Rediscovery of *R.hilli* and documenting new records of *Rhinolophuslanderi* and *Kerivoulaargentata* reinforces the universal value of Rwanda's committed stewardship of Nyungwe National Park as a global biodiversity hotspot.

By recording the first echolocation signature for *R.hilli*, we provide a beneficial tool for conservation managers to conduct cost-effective monitoring that provides information for conservation planning. The on-going acoustic monitoring conducted by Nyungwe National Park staff continues to identify the core range of the species within the Park, which greatly improves knowledge about critical habitat needs of the species. Furthermore, by contributing the echolocation signature to the ChiroVox global acoustic call database (Görföl et al. 2022), we aid any future acoustic monitoring projects conducted by researchers and conservation managers working in the Albertine Rift region of Central Africa.

## Project description

### Study area description

Bat surveys were conducted in Nyungwe National Park in south-western Rwanda and in a few locations near the park boundary. Sampling locations within Nyungwe National Park were primarily caves and forest trails. Surveys for bats were conducted within the Uwinka region of Nyungwe and in similar habitats in the Park to determine the presence of an extant population of *R.hilli* and document all bat species encountered.

### Design description

**Figure 1.**
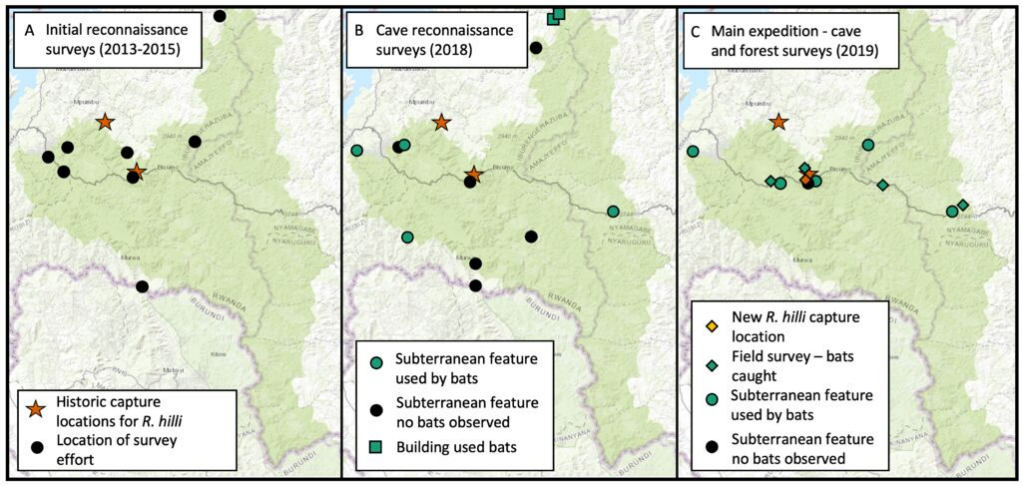
Location of survey efforts undertaken to rediscover *Rhinolophushilli* in Nyungwe National Park, Rwanda. **A** Initial reconnaissance surveys (2013-2015); **B** Cave reconnaissance surveys (2018); **C** main expedition cave and forest surveys (2019).

Surveys were conducted in four phases: Initial reconnaissance surveys (2013-2015); reconnaissance for cave suitability by Nyungwe National Park Rangers (2018); a survey expedition with trapping efforts in forest habitats, cave surveys to assess bat use, and acoustic sampling of bat echolocation activity (2019); and on-going acoustic monitoring conducted by Nyungwe National Park Rangers (2019-present). Initial reconnaissance surveys were carried out by a small team (P. Webala and J. Nziza) with the intention of determining if the presence of *R.hilli* in Nyungwe National Park was readily detectable with minimal survey effort. Surveys were targeted in the Uwinka region of Nyungwe National Park and surrounding areas where bat roosts had been reported. Over the course of two years, 10 sites were surveyed with eight species detected (Fig. 1A). However, *R.hilli* was not detected during these rapid assessment surveys.

In the cave suitability reconnaissance phase, Nyungwe National Park Rangers identified caves within Nyungwe National Park with features suitable for bat occupancy. We provided Nyungwe National Park Rangers with a pictorial cave survey form to describe the size, type, and location of caves and abandoned mines and to report on any sign of bats using subterranean features. Rangers reported caves encountered during patrols and queried local communities to identify sites. Rangers identified and located a total of ten caves, one abandoned mine, and one building as potential bat roost habitats prior to the planned survey expedition in early 2019 (Fig. 1B).

**Figure 2.**
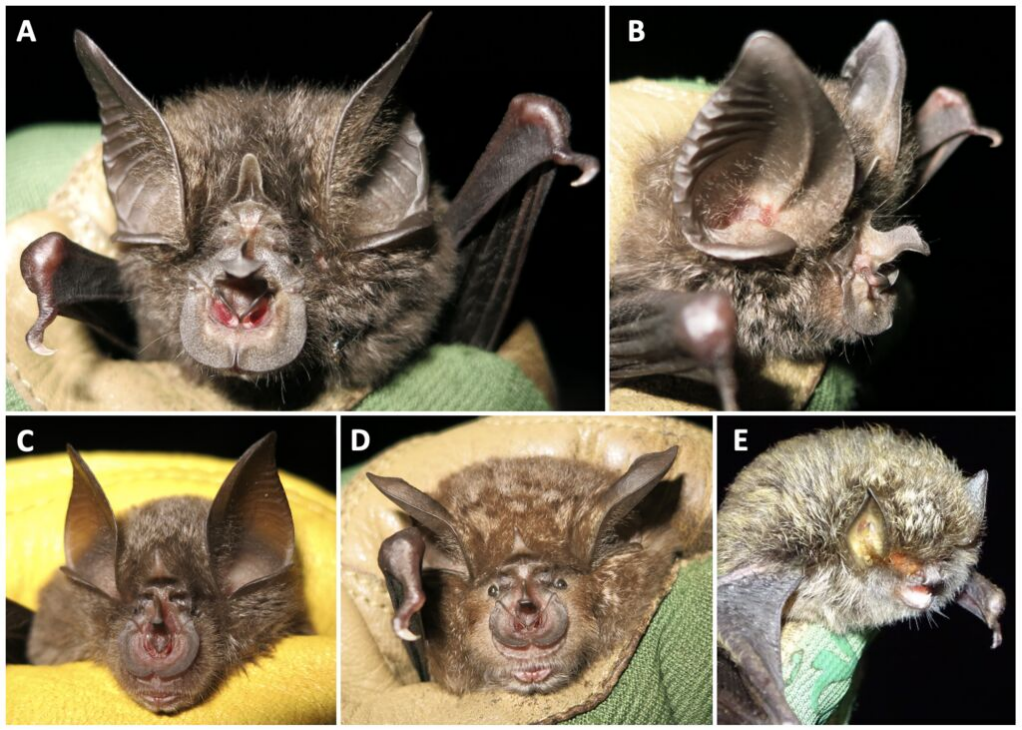
Notable records of bat species encountered in Nyungwe National Park. **A, B** *Rhinolophushilli*, first observation of this species since 1981; **C** *Rhinolophuslanderi*, first record of this species in Nyungwe National Park; **D** *Rhinolophusclivosus*, congeneric species found in Nyungwe for comparison; **E** *Kerivoulaargentata*, first record of this species in Rwanda.

**Figure 3.**
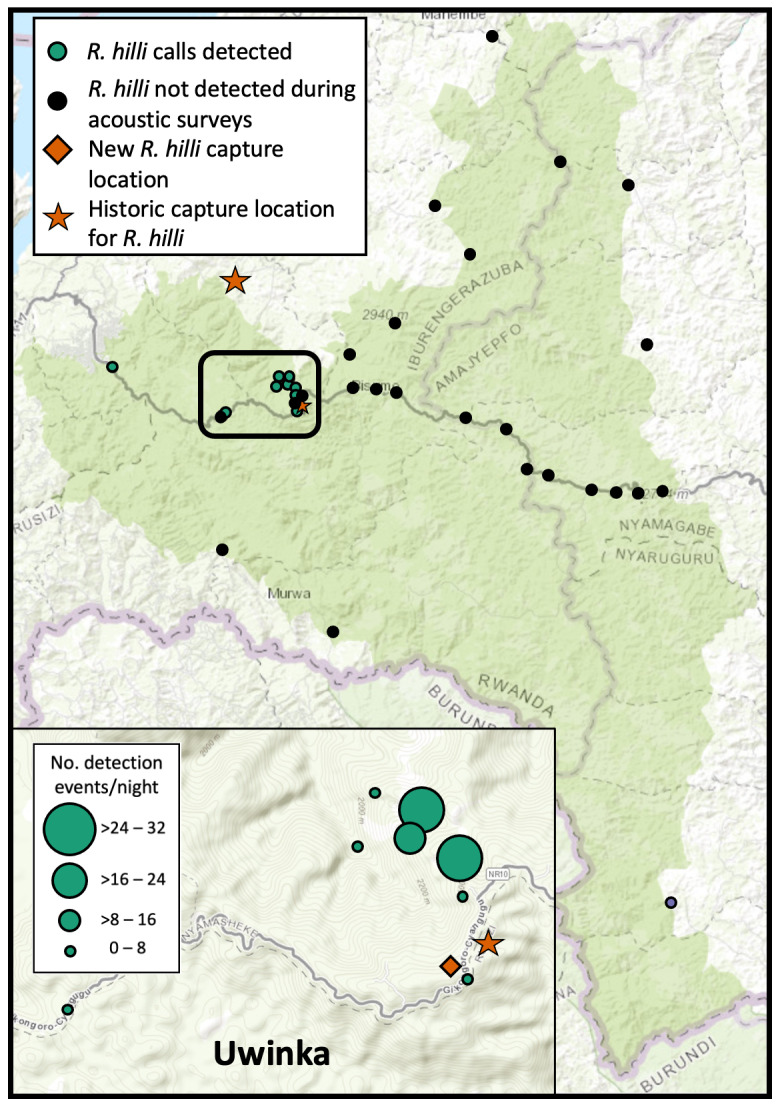
Location of acoustic surveys for *Rhinolophushilli* conducted from July 2019 to November 2020 in Nyungwe National Park, Rwanda. Locations where *R.hilli* were detected and frequency in which *R.hilli* calls were identified (measured by number of positive triggering events/night, inset) are shown.

We conducted a 10-day intensive field survey from 13-23 January 2019 that focused on: (1) surveys for bat use at caves identified by Nyungwe Rangers as suitable and likely to be occupied by bats, (2) capture surveys in forested habitats in the Uwinka region and similar surrounding habitats in Nyungwe National Park, and (3) acoustic sampling of bat echolocation activity using SongMeter 4BAT-FS recorders (Wildlife Acoustics, Inc). In total, 17 locations were surveyed, ten within the Nyungwe National Park boundary (Fig. 1C), with 55 bats from five families caught, including Hill's horseshoe bat (*Rhinolophushilli)*, Lander's horseshoe bat (*Rhinolophuslanderi*) and the Damara woolly bat (*Kerivoulaargentata*) (Fig. 2, Table 1, Suppl. material 1). This survey effort was scheduled to occur during the short dry season as the first of several planned survey trips intended to sample in the dry and wet seasons to determine the seasonality of bat occurrences. Future survey expeditions have been delayed until further notice due to safety precautions and travel restrictions during the COVID-19 pandemic. Since 2019, Nyungwe National Park Rangers have conducted acoustic sampling within the Park as part of a long-term bat acoustic monitoring project using two SongMeter 4BAT-FS recorders. With this dataset, we report the acoustic detections of *R.hilli*, *R.landeri*, and *R.clivosus* resulting from sampling effort at 35 locations within the Park over a total of 166 nights from July 2019 through November 2020 (Fig. 3).

## Sampling methods

### Study extent

Survey efforts focused within Nyungwe National Park and surrounding areas in south-western Rwanda. The dataset includes 278 occurrence records from 10 bat species of five families detected at 71 locations in or near Nyungwe National Park.

### Sampling description

**Cave surveys**: We surveyed caves by visually searching with the aid of bright lights all accessible areas for the presence of bats or signs of bat use. We noted the presence of bat guano or wall staining, if present. At sites with areas inaccessible to human observers, we deployed acoustic detectors (SongMeter 4BAT-FS with SMM-U2 ultrasonic microphones, Wildlife Acoustics, Inc.) at entrances for 1-2 nights and used Kaleidoscope Pro (version 5.4.2, Wildlife Acoustics, Inc.) to identify the presence of bat echolocation activity during crepuscular and nocturnal hours. If bats were present during an internal search, we captured bats with hand nets or placed harp traps (details below) at the entrance prior to evening emergence.

**Capture surveys in forest habitats**: Capture surveys were conducted with harp traps (a 2-bank 4.2 m^2^ harp trap by Ausbat and the ‘cave-catcher’ 2-bank 0.9 m^2^ harp trap by Bat Conservation and Management) and use of three to five mist-nets of 2 m, 6 m and 12 m lengths (Avinet). We placed harp traps and mist-nets parallel or perpendicular to forest trails in locations selected to maximise capture probability. Harp traps were deployed from sunset until sunrise. We opened mist-nets at sunset and monitored for approximately 4 hours and then re-opened 1-2 hours before sunrise. We monitored mist-nets continuously while open every 10-15 mins. We held bats individually in clean, cloth bags until processed and then released bats at the location of capture. See ‘Step Description’ for the description of data collected from captured bats.

**Acoustic sampling**: Nyungwe Park Rangers deployed SongMeter 4BAT-FS acoustic recorders (Wildlife Acoustics, Inc) at locations along forest trails or near cave entrances during multi-day patrols and collected recorders when returning from patrol. The SM4BAT-FS recorders with SMM-U2 microphones were programmed to record in full-spectrum at 384 kilohertz sampling frequency with 12 dB gain and 16 k high filter. All other settings were set to default. The SM4BATs were set to record 30 minutes before sunset to 30 minutes after sunrise and were typically deployed for 3-5 nights at each location. We embedded geo-location coordinates on all files using the GPS attachment available from Wildlife Acoustics, Inc. Data were transferred to external hard drives and sent to Bat Conservation International in the USA for processing. See ‘Step Description’ for the description of the processing of acoustic data for species identification of *R.hilli*.

### Quality control

For a subset of tissue samples, we compared species identification determined from morphological measurements with genetic data using BLASTN. As we were unable to obtain viable DNA from the holotype *R.hilli* specimen collected in 1981, we inspected both museum samples and compared morphological features with measurements of the two *R.hilli* caught during our survey. In addition, we compared the sequence data from the two suspected *R.hilli* samples with sequence data from closely-related species (Demos et al. 2019) to confirm that our classification was accurate.

### Step description

**Species Identification and Morphometrics**: We assessed captured bats for age (juvenile/sub-adult/adult), sex, and reproductive condition (females: non-breeding/pregnant/lactating/post-lactating; males: reproductively active/non-reproductively active as determined by enlarged testes) (Racey 2009). We measured standard morphometrics, including forearm length, tibia length, hind-foot length, tail length, ear length, tragus length, body length, and mass. We used the Mammals of Africa Volume IV (Hedgehogs, Shrews and Bats) (Kingdon 2013) as the primary key for species identification. We sampled skin tissue using a 3-mm biopsy punch from the wing membrane and stored skin tissue in desiccant until the DNA was extracted.

**Figure 4.**
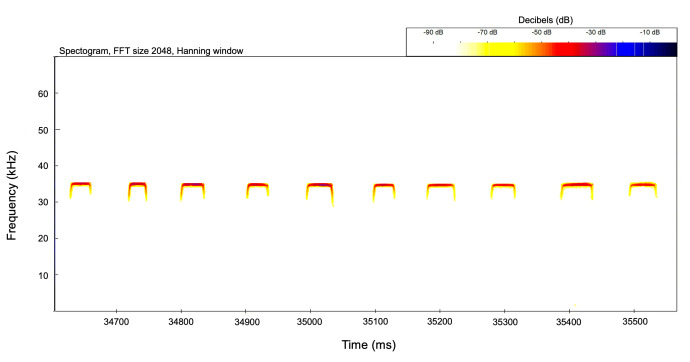
Spectrogram of echolocation calls emitted by *Rhinolophushilli* (FFT size 2048, Hanning window; sampling rate of 500 kHz). Color scale represents amplitude of sound in decibels (dB). Mean peak frequency 35.4 kHz, call duration 49.5 ms, interpulse interval 82.25 ms.

**Echolocation Voucher Calls**: We recorded voucher echolocation calls upon release for each echolocating bat species using an M500 full-spectrum bat detector (Pettersson Electronics) at a sampling rate of 500 kHz. For constant-frequency (CF) bats (e.g. *Rhinolophus* spp.), we recorded resting echolocation calls while the bat was in hand. For species using frequency-modulated (FM) echolocation, we recorded echolocation activity in flight immediately upon release while visually following the bat with a light. Hand-recorded bat echolocations were analysed using BatSound v.4.1 (Pettersson Electronics) to determine the following parameters for each pulse: duration (D), maximum frequency (FMAX), minimum frequency (FMIN), peak frequency (PF), and interpulse interval (IPI). We measured these parameters (D, FMAX, FMIN, and IPI) from spectrograms and the peak frequency (PF) from the power spectrum. We removed noise files and filtered the remaining files for constant frequency acoustic signatures (>15 ms call duration) using Kaleidoscope Pro (version 5.4.2, Wildlife Acoustics). Echolocation calls matching those of voucher calls collected from *R.hilli* (*Fig. 4*), *R.landeri*, and *R.clivosus* were identified during call analysis. All data are preserved to allow for future analysis once other call signatures are identified. Voucher calls will be contributed to the ChiroVox database (www.chirovox.org; Görföl et al. 2022).

**DNA Extraction for Species Confirmation**: DNA extraction from wing biopsy punches was carried out at CIBIO-InBIO, University of Porto, Portugal, using Qiagen DNeasy kits (Qiagen, Crawley, UK) and stored at -20^o^C. Mitochondrial cytochrome b (cyt b) gene was amplified by polymerase chain reaction (PCR) using the primers MOLCIT‐F (5′-AATGACAT-GAAAAATCACCGTTGT-3′) (Ibáñez et al. 2006) and MVZ16-R (5′-AAATAGGAARTATCAYTCTGGTTTRAT-3′) (SMITH and PATTON 1993). PCRs were performed in a 10 μl volume, which included 1 μl of DNA extract, 0.4 μl of each primer (10 μM), 5 μl of Qiagen Master Mix, and double-distilled water was added until final volume was reached. Reactions were performed under the following conditions: 95^o^C for 15 min; 40 cycles of 95^o^C for 30 s, 50^o^C for 45 s, 72^o^C for 1 min; 60^o^C for 10 min, and DNA sequencing performed on an ABI3700 DNA sequencer (Applied Biosystems). Chromatograms were edited aligned using Mega X (Kumar et al. 2018) with sequences (OK377601-OK377653) submitted using a via Standard Nucleotide BLAST search on the NCBI website . For phylogenetic comparison, edges of incomplete sequences were trimmed to reduce missing data. Models of sequence evolution were explored in jModel test v.2.1.10 (Darriba et al. 2012) using the Bayesian Information Criterion (BIC). Bayesian Inference (BI) was performed using MrBayes v.3.2.7 (Huelsenbeck and Ronquist 2001, Ronquist and Huelsenbeck 2003). BI trees were run with four simultaneous chains, each of 1×10^7^ generations, sampled every 1000 generations, and with the first 25% of trees discarded as burn-in. Convergence was assessed using effective sampling size in Tracer v.1.7.1 (Rambaut et al. 2018).

**Figure 5.**
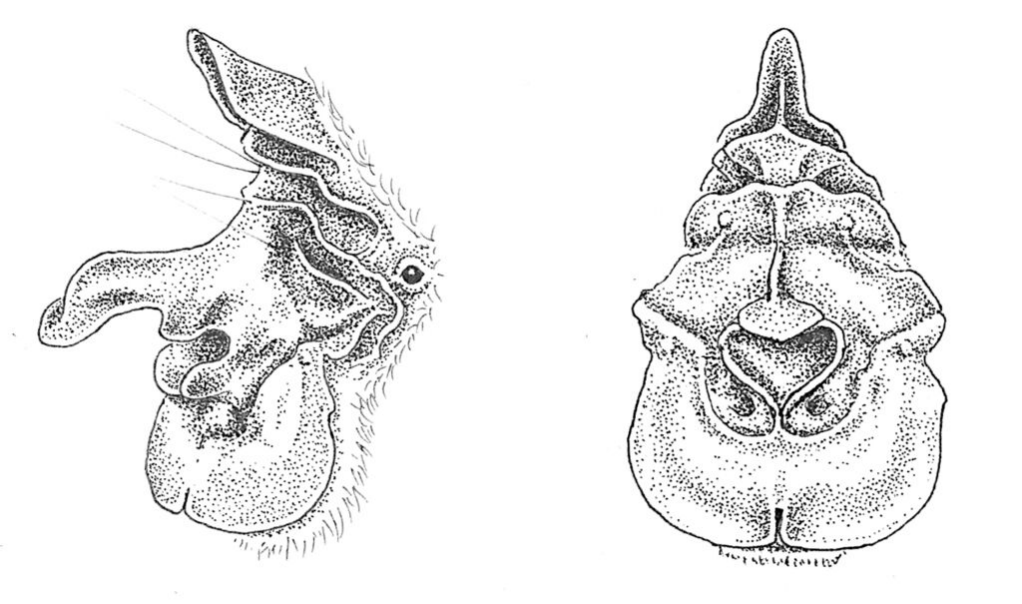
Sketches of noseleaf morphology of *Rhinolophushilli* from photographs of the two individuals captured during this study. Drawings by Fiona Reid.

**Figure 6.**
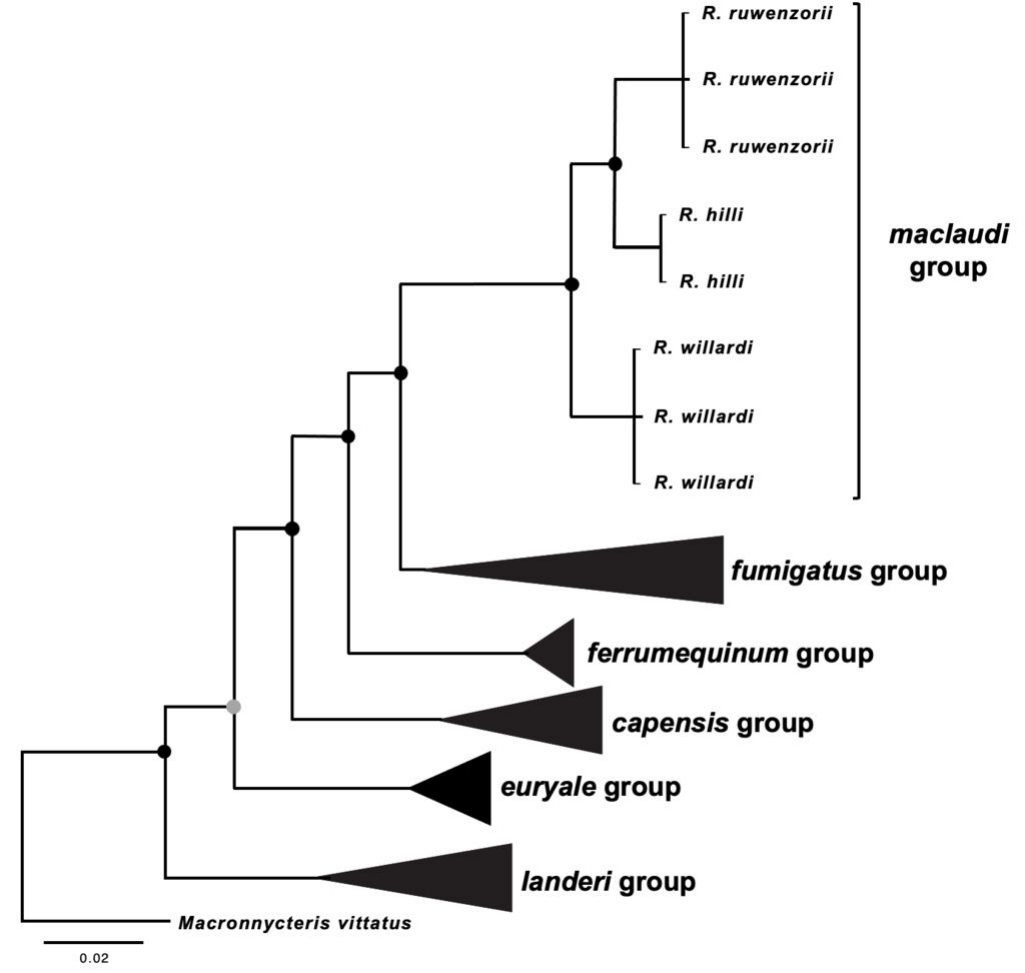
Bayesian phylogeny of selected indivduals from the genus *Rhinolophus* using an alignment of 490 base pairs of mitochondrial gene cytochrome b. Black circles at nodes represent Bayesian posterior probabilities (PP) ≥ 0.95, and grey circles represent PP < 0.95. Tip labels represent major clades and more detailed breakdown of species names for *R.maclaudi* group. Species used in the phylogenetic analysis, including GenBank accession numbers are outlined in Suppl. material 2.

Due to the age and preservation method, we were unable to obtain viable DNA from the two museum specimens of *R.hilli* . Instead, to verify species identification, we inspected both specimens and compared morphological features with measurements of the two *R.hilli* caught during our survey (Table 2, Fig. 5). In addition, we compared the sequence data from the two suspected *R.hilli* samples with sequence data from closely-related species to confirm that our classification was accurate (Fig. 6). For the remaining species, we compared species identification determined from morphological measurements with genetic data, using BLASTN for a subset of tissue samples.

## Geographic coverage

### Description

Nyungwe National Park is the second-largest national park in Rwanda, protecting 1,019 km^2^ of Afromontane rainforest habitat in the Albertine Rift region of Africa. The Park is recognised for exceptionally high biodiversity with 1,068 recorded plant species, 322 bird species and 75 known mammal species, including 13 primates (African Parks 2021). Nyungwe National Park has been managed by the Nyungwe Management Company under African Parks in a management agreement with the Rwanda Development Board since October 2020.

### Coordinates

-2.918 and -2.142 Latitude; 28.869 and 29.575 Longitude.

## Taxonomic coverage

### Description

The dataset includes occurrence records from Class Mammalia and Order Chiroptera, including 13 taxonomic records representing 10 genera and five families. Three records were identified to genus with the remaining 10 identified to species.

### Taxa included

**Table:** 

Rank	Scientific Name	Common Name
class	Mammalia	Mammals
order	Chiroptera	Bats
family	Rhinolophidae	Horseshoe Bats
family	Hipposideridae	Roundleaf Bats
family	Nycteridae	Slit-faced Bats
family	Pteropodidae	Old World Fruit Bats
family	Vespertilionidae	Vesper Bats

## Temporal coverage

### Notes

2013-05-10 through 2020-11-14

## Usage licence

### Usage licence

Creative Commons Public Domain Waiver (CC-Zero)

## Data resources

### Data package title

Bat species occurrences in Nyungwe National Park, Rwanda

### Resource link

https://doi.org/10.15468/k24rd6

### Alternative identifiers

http://ipt.vertnet.org:8080/ipt/resource?r=bci_rwanda&v=1.6

### Number of data sets

1

### Data set 1.

#### Data set name

Bat species occurrences in Nyungwe National Park, Rwanda

#### Data format

Darwin Core

#### Download URL

https://www.gbif.org/dataset/e9bf7d9e-8b31-4a02-8203-f7153b5d64c6

#### Description

The dataset includes bat species occurrence records resulting from survey efforts in Nyungwe National Park and surrounding areas in south-western Rwanda from 2013 to 2020 (Flanders et al. 2022). Data were collected as part of a long-term collaborative project to determine if the IUCN critically-endangered Hill's horseshoe bat (*Rhinolophushilli*) is extant. The dataset includes species occurrence records resulting from cave roost surveys, capture surveys, and acoustic sampling of bat echolocation activity. The dataset includes 278 occurrence records from 10 bat species of five families detected at 71 locations in or near Nyungwe National Park. The dataset includes three notable species occurrences in Nyungwe National Park, including the first detection of Hill's horseshoe bat (*Rhinolophushilli*) since 1981, the first record of Lander's horseshoe bat (*Rhinolophuslanderi*) in Nyungwe National Park, and the first record of the Damara woolly bat (*Kerivoulaargentata*) in Rwanda.

**Data set 1.** 

Column label	Column description
Scientific name	Scientific name (Genus and species)
Country or Area	Country name where observation occurred
Coordinates	Lat/Long
Month and Year	Month and Year when observation occurred
Basis of record	Method of observation
Dataset	Name of dataset
Kingdom	Kingdom
Phylum	Phylum
Class	Class
Order	Order
Family	Family
Genus	Genus
Species	Species

## Supplementary Material

5D1ACB6E-612C-5370-B349-7B57372A9837 10.3897/BDJ.10.e83546.suppl1 Supplementary material 1 Occurrence data with associated GenBank accession numbers. Data type Species description, occurrence, and GenBank accession numbers. File: oo_637313.xlsx https://binary.pensoft.net/file/637313 Flanders J, Frick W, Nziza J, Nsengimana O, Kaleme P, Dusabe M C, Ndikubwimana I, Twizeyimana I, Kbiwot S, Ntihemuka P, Cheng T, Muvunyi R, Webala P.

BB1F61C1-9C2A-5423-B3F6-176E661F583C 10.3897/BDJ.10.e83546.suppl2 Supplementary material 2 Species information, including GenBank accession numbers, used for phylogenetic analysis. Data type Phylogenetic File: oo_680360.xlsx https://binary.pensoft.net/file/680360 Flanders J, Frick W, Nziza J, Nsengimana O, Kaleme P, Dusabe M C, Ndikubwimana I, Twizeyimana I, Kbiwot S, Ntihemuka P, Cheng T, Muvunyi R, Webala P.

## Figures and Tables

**Table 1.** Bat species encountered in Nyungwe National Park and surrounding areas from 2013-2020, their IUCN Red List Status and the habitat type where observations occurred.

Taxon	IUCN Red List Status	Occurrence Habitat
**Hipposideridae**		
*Hipposideroscaffer*	Least Concern	Cave/Mine/Forest
*Hipposiderosruber*	Least Concern	Building
**Nycteridae**		
*Nycteris* sp.		Cave/Mine
**Pteropodidae**		
*Epomophoruslabiatus*	Least Concern	Village
*Myonycterisangolensis*	Least Concern	Cave/Forest
*Rousettusaegyptiacus*	Least Concern	Cave/Forest
*Stenonycterislanosus*	Least Concern	Cave/Forest
**Rhinolophidae**		
*Rhinolophusclivosus*	Least Concern	Mine/Forest
*Rhinolophushilli* ^1^	Critically Endangered	Forest
*Rhinolophuslanderi* ^2^	Least Concern	Cave/Forest
**Vespertilionidae**		
*Kerivoulaargentata* ^3^	Least Concern	Forest
*Miniopterus* sp.		Forest
*Neoromicia* sp.		Forest

^1^First record since 1981; ^2^First record in Nyungwe National Park; ^3^First record in Rwanda.

**Table 2.** Morphological measurements from two *Rhinolophushilli* captured in 2019 (BCI RW19009, RW19052) compared to those recorded by Fahr et al. 2002) for the holotype (Zoologisches Museum der Universität Zürich, Switzerland; ZMUZ 126639) and the other preserved specimen (Musée Royal de l'Afrique Centrale, Belgium; MRAC 82006M1) of this species.

Sample Number	BCI RW19009	BCI RW19052	ZMUZ 126639	MRAC 82006M1
Sex	M	M	F	F
Age	Adult	Adult	Adult	Adult
Mass (grams)	14.0	15.0	-	16.5
Forearm length (mm)	53.35	49.75	54.3	54.2
Tail length (mm)	21.10	26.55	29.3	-
Hind-foot length (mm)	8.80	9.50	12.2	-
Ear length (mm)	23.25	24.33	28.5	-
Horseshoe-width (mm)	13.25	14.35	-	-
Tibia length (mm)	24.3	21.1	23.8	-
4th Metacarpal length (mm)	-	-	40.9	40.6
4th Phalange length (mm)	-	-	10.9	10.6
